# Beyond the triglyceride-glucose index, the cholesterol- high-density lipoprotein -glucose index as a superior predictor for diabetes risk in patients with major adverse cardiovascular events: dual evidence from the CHARLS database and real-world data

**DOI:** 10.3389/fendo.2026.1797342

**Published:** 2026-03-12

**Authors:** Tao Sun, Hui Yan, Mixue Guo, Jun Liu, Wei Zhao

**Affiliations:** 1 School of Basic Medicine, Ningxia Medical University, Yinchuan, China; 2 Department of Hematology and Oncology Laboratory, The Central Hospital of Shaoyang, Shaoyang, Hunan, China; 3 Ningxia Key Laboratory of Prevention and Treatment of Common Infectious Diseases, Ningxia Medical University, Yinchuan, China; 4 Department of Scientific Research, The First Affiliated Hospital of Shaoyang University, Shaoyang, Hunan, China

**Keywords:** cholesterol-HDL-glucose index (CHG Index), cohort study, diabetes risk, insulin resistance, major adverse cardiovascular events, risk prediction, triglyceride-glucose index (TyG Index)

## Abstract

**Background:**

To compare the predictive value of the cholesterol-high-density lipoprotein-glucose (CHG) index versus the triglyceride-glucose (TyG) index for new-onset diabetes in patients with major adverse cardiovascular events (MACE) using two independent cohorts.

**Methods:**

This multicenter study enrolled 1,138 patients (median follow-up: 7.99 years) from the China Health and Retirement Longitudinal Study (CHARLS) cohort and 614 patients (median follow-up: 2.61 years) from the Central Hospital of Shaoyang (CHSY) cohort. Multivariable Cox regression, Kaplan-Meier curves, restricted cubic spline (RCS) analysis, subgroup analysis, and sensitivity analysis were employed to assess associations. Predictive performance was compared using receiver operating characteristic (ROC) curves and machine learning.

**Results:**

New-onset diabetes occurred in 181 (15.9%) and 71 (11.6%) patients in the CHARLS and CHSY cohorts, respectively. After adjustment, the hazard ratios (HRs) per 1-standard deviation increase in the CHG index were 1.32 (95% CI: 1.14–1.53) and 1.63 (1.33–2.02) in the two cohorts, respectively. The corresponding HRs for the TyG index were 1.30 (1.12–1.50) and 1.50 (1.22–1.85). Both indices showed a linear positive association (RCS P for nonlinearity >0.05). The CHG index demonstrated a slightly higher area under the curve (AUC) value in the CHARLS study (0.632 vs. 0.626) and greater feature importance in machine learning models.

**Conclusions:**

Both the CHG and TyG indices independently predict the risk of diabetes in patients with MACE. However, the CHG demonstrates a slight advantage in overall predictive accuracy and model calibration, suggesting it may be a better metabolic indicator for assessing the risk of glucose metabolism disorders following cardiovascular events.

## 1 Introduction

Cardiovascular disease (CVD) and diabetes mellitus (DM) are mutually profound risk factors, both imposing a substantial disease burden worldwide (1). Epidemiological studies have demonstrated that patients with diabetes have a significantly elevated risk of major adverse cardiovascular events (MACE) (2–4); conversely, the incidence of new-onset diabetes is markedly higher among MACE survivors compared to the general population (5, 6). This high-risk state of “dysglycemia following cardiovascular events” not only increases the probability of recurrent cardiovascular events and mortality but also poses significant challenges for clinical secondary prevention (7, 8). Therefore, exploring effective metabolic predictors for the early identification of individuals at high risk for diabetes among MACE patients holds considerable clinical and public health significance.

Insulin resistance (IR) is a core pathological process in the development and progression of diabetes and also serves as a critical metabolic basis for atherosclerosis and cardiovascular events (9, 10). The traditional assessment of insulin resistance relies on the homeostasis model assessment of insulin resistance (HOMA-IR) or the hyperinsulinemic-euglycemic clamp technique. However, these methods are complex, costly, and challenging to implement in large populations (11). In recent years, simplified metabolic indices derived from routine metabolic parameters have attracted increasing attention in epidemiological and clinical research (12–14). Among them, the triglyceride-glucose (TyG) index is widely used as a surrogate for HOMA-IR and is closely associated with the risk of diabetes, metabolic syndrome, and cardiovascular events (15–17). Nevertheless, the TyG index primarily reflects the interaction between triglycerides (TG) and fasting plasma glucose (FPG), failing to fully account for the important roles of cholesterol metabolism and lipoprotein structural abnormalities in glucose and lipid metabolic disorders.

Consequently, the cholesterol-HDL-glucose (CHG) index has been proposed, which integrates total cholesterol (TC), high-density lipoprotein cholesterol (HDL-C), and FPG levels, thereby providing a more comprehensive reflection of the interplay between lipid and glucose metabolism (18–21). Previous studies have suggested that the CHG index is significantly associated with insulin resistance, non-alcoholic fatty liver disease, and atherosclerosis risk. However, its value in predicting new-onset diabetes in MACE patients remains unclear, with limited validation from prospective, multicenter studies.

Therefore, utilizing two large population cohorts—the China Health and Retirement Longitudinal Study (CHARLS) and the clinical database of the Central Hospital of Shaoyang (CHSY)—this study systematically evaluated the predictive performance of the CHG and TyG indices for the risk of new-onset diabetes in MACE patients. Through multivariable Cox regression models, restricted cubic spline (RCS) analysis, stratified validation across multiple subgroups, and incorporation of machine learning methods (Random Forest and SHapley Additive exPlanations (SHAP) analysis), this study aims to: (1) determine the independent associations of the CHG and TyG indices with the risk of new-onset diabetes in MACE patients; (2) compare the predictive capabilities of these two indices; and (3) explore the potential clinical application value of the CHG index in assessing the risk of dysglycemia following cardiovascular events.

## 2 Materials and methods

### 2.1 Study design and data sources

This study was a multicenter cohort study based on the CHARLS database and the clinical database of CHSY. The CHARLS database used 2011 as the baseline, with follow-up until 2020, a maximum follow-up duration of 9 years, and a median follow-up period of 7.99 years (22, 23). The CHSY database utilized clinical data from 2013–2022 as the baseline, with a minimum continuous follow-up of 2 years, a maximum continuous follow-up of 8 years (until 2024), and a median follow-up period of 2.61 years. The primary exposure factors were CHG and TyG. The primary outcome event was the new-onset diabetes during the follow-up period in survivors who had experienced MACE. The incidence of new-onset diabetes was defined as the first occurrence of any of the following conditions during follow-up among participants without diabetes at baseline: (1) FPG ≥126 mg/dL; (2) self-reported physician diagnosis (applied to the CHARLS cohort); (3) a new prescription for anti-hyperglycemic medications; or (4) a discharge diagnosis of diabetes (applied to the CHSY cohort). In the CHARLS cohort, diagnoses were primarily confirmed through biennial follow-up surveys and concurrent FPG measurements. In the CHSY cohort, diagnoses were confirmed via annual follow-up, electronic medical record review, and laboratory testing. The diagnosis date was defined as the earliest date on which any diagnostic criterion was met.

### 2.2 Study participants

**Figure 1:**
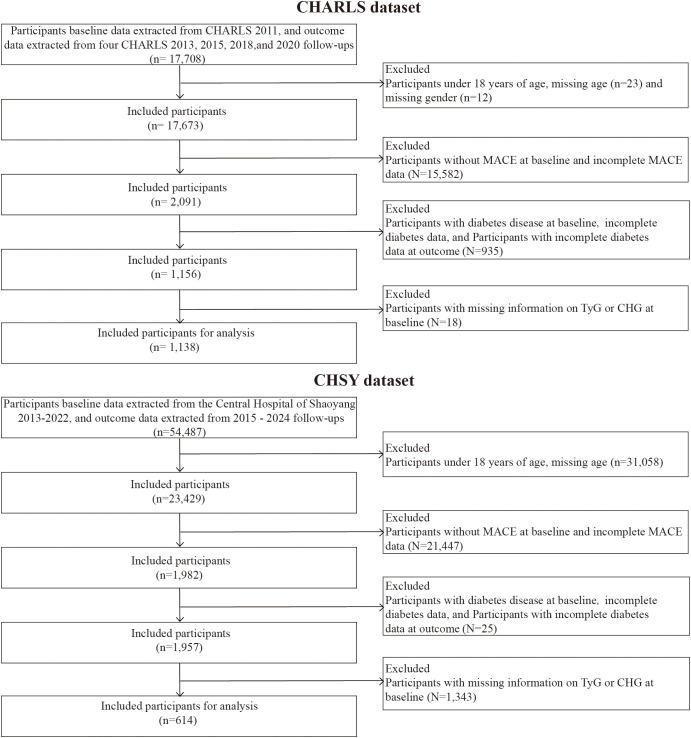
Flow chart of the selection of study participants.

A total of 17,708 participants were initially included in the CHARLS database at baseline. After screening according to the inclusion and exclusion criteria, 1,138 eligible subjects were ultimately included. The CHSY database initially recorded 54,487 patients at baseline; after screening using the same criteria, 614 subjects were included. Inclusion criteria: (1) Age ≥18 years; (2) A documented history of major adverse cardiovascular events (MACE) at baseline, defined as patients with myocardial infarction, acute coronary syndrome or ischemic heart disease (ACS/IHD), stroke (ischemic or hemorrhagic stroke), or revascularization procedures; (3) Availability of complete baseline clinical and laboratory data; (4) Follow-up duration of not less than 2 years. Exclusion criteria: (1) Pre-existing diagnosis of diabetes at baseline (including previous medical history or current use of glucose-lowering therapy); (2) Lack of key laboratory measurements such as fasting plasma glucose and blood lipids; (3) Severe data deficiency during follow-up or inability to determine the occurrence of the endpoint event. The patient screening flowchart is shown in **Figure 1**.

### 2.3 Primary exposure variables and covariates

The primary exposure variables were the CHG and TyG indices. The TyG index was calculated using the following formula (24, 25):


$$\text{TyG}=\ln\left(\frac{\text{TG (mg/dL)}\times \text{FPG (mg/dL)}}{2}\right)$$

The CHG index was calculated based on TC, HDL-C, and FPG, using a standardized formula reported in previous literature (20):


$$\text{CHG}=\ln\left(\frac{\text{TC (mg/dL)}\times \text{FPG (mg/dL)}}{\text{HDL-C (mg/dL)}}\right)$$

Where TC denotes total cholesterol (mg/dL), TG denotes triglycerides (mg/dL), HDL-C denotes high-density lipoprotein cholesterol (mg/dL), and FPG denotes fasting plasma glucose (mg/dL). Note on Units of Measurement: In the CHARLS cohort, all original lipid and glucose values were recorded in mg/dL and were used directly in calculations. In the CHSY cohort, the original units for lipids and glucose were mmol/L, and the original unit for uric acid (UA) was μmol/L; prior to index calculation, these values were converted to mg/dL using standard conversion factors (TG: ×88.57; TC, HDL-C, LDL-C: ×38.66; FPG: ×18.02; UA: ×0.0168). In the CHARLS dataset, TC, TG, LDL-C, HDL-C, uric acid (UA), and FPG were determined by the Youanmen Centre for Clinical Laboratory at Capital Medical University. In the CHSY dataset, these indicators were measured using an AU5800 biochemical analyzer by laboratory personnel in the Department of Clinical Laboratory at the Central Hospital of Shaoyang. Covariates included demographic characteristics (age, sex, marital status, type of household registration), lifestyle factors (smoking, drinking), history of chronic diseases (hypertension, malignant tumors, lung diseases, liver diseases, kidney diseases, arthritis or rheumatism), and biochemical indicators (BMI, TC, TG, LDL-C, HDL-C, UA, FPG). Definitions of all variables are provided in **Supplementary Table 1**.

### 2.4 Statistical analysis

All statistical analyses were performed using R software (version 4.2.2) and Python software (version 3.11.5). The distribution of continuous variables was assessed using the Lilliefors (Kolmogorov–Smirnov) test (**Supplementary Table 2**) (26). Normally distributed data were presented as mean ± standard deviation and compared between groups using the independent-samples t-test. Non-normally distributed data are presented as median (interquartile range) and were compared using the Wilcoxon rank-sum test. Categorical variables were presented as frequencies (percentages) and compared between groups using the χ² test or Fisher’s exact test. For variables with a low proportion of missing data in the baseline characteristics (missing rate<30%), multiple imputation was used to handle missing values (27). Correlation and multicollinearity analyses were conducted among variables. The proportional hazards assumption for the Cox regression models was tested using Schoenfeld residuals (28). The primary analysis utilized Cox proportional hazards regression models. Three models were sequentially constructed: Model 1 was unadjusted for covariates; Model 2 was adjusted for age, sex, smoking status, alcohol consumption, and body mass index (BMI); Model 3 was further adjusted for history of various chronic disorders based on Model 2. Results are expressed as hazard ratios (HRs) with 95% confidence intervals (CIs). The cumulative incidence of diabetes mellitus among patients with MACE was estimated using Kaplan-Meier (KM) survival curves, and the log-rank test was used to assess differences. To explore potential nonlinear relationships between the TyG index, the CHG index, and the risk of incident diabetes, RCS were fitted. Stratified analyses were performed across subgroups defined by clinical characteristics, including sex, age, and hypertension status. Given the imbalanced distribution between the diabetic and non-diabetic groups, the Synthetic Minority Over-sampling Technique (SMOTE) was employed within the classification framework as a sensitivity analysis to evaluate the robustness of the discriminative performance of each indicator (29, 30). Furthermore, receiver operating characteristic (ROC) curves were plotted for both the original and SMOTE-balanced datasets to compare the predictive capabilities of the TyG index, CHG index, and their combination for diabetes onset; the area under the curve (AUC) was calculated for each. Finally, a Random Forest model was utilized to rank variable importance (31), and the SHAP model was employed to interpret the contribution of variables to diabetes risk (32). All statistical tests were two-sided, and P< 0.05 was considered statistically significant.

## 3 Results

### 3.1 Baseline characteristics and data quality

In both the CHARLS and CHSY datasets, the missing rates for most variables were below 10%. Only afew variables had slightly higher rates, but all were<30%, meeting the criteria for multiple imputation (**Supplementary Figures 1A, B**). The variable distributions were highly consistent before and after imputation, indicatingno significant bias was introduced (**Supplementary Figures 1C, D**). Correlation analyses revealed moderate to strong positive correlations among metabolic indicators such as TyG, CHG, TG, and FPG. In contrast, demographic characteristics and variables related to past medical history showed weak correlations with TyG and CHG (**Supplementary Figures 2A, B**). The variance inflation factor (VIF) for all variables was<10, indicating no significant multicollinearity (**Supplementary Figures 3A, B**).

A total of 1138 patients who experienced major adverse cardiovascular events (MACE) were included from the CHARLS dataset, among whom 181 (15.9%) developed incident diabetes during follow-up. Compared to the non-diabetes group, the diabetes group had significantly higher levels of body mass index (BMI), TG, uric acid (UA), FPG, TyG, and CHG, as well as a higher prevalence of hypertension and malignant tumors (**Table 1**). In the CHSY dataset, 614 MACE patients were included, with 71 (11.6%) developing incident diabetes. The TyG and CHG levels were significantly higher in the diabetes group compared to the non-diabetes group, and the prevalence of hypertension was also higher (**Supplementary Table 3**). The overall trends were consistent with those observed in the CHARLS dataset.

**Table 1:** Patient demographics and baseline characteristics in the CHARLS dataset.

Characteristic	Event			p-value
	Overall N = 1,138	MACE with diabetes N = 181	MACE without diabetes N = 957	
Age, Median (Q1, Q3)	61.00 (54.00, 68.00)	60.00 (54.00, 66.00)	61.00 (55.00, 69.00)	0.100
Gender, n (%)				0.560
Female	682 (59.93%)	112 (61.88%)	570 (59.56%)	
Male	456 (40.07%)	69 (38.12%)	387 (40.44%)	
Marital, n (%)				0.411
Divorced	16 (1.41%)	2 (1.10%)	14 (1.46%)	
Married	951 (83.57%)	158 (87.29%)	793 (82.86%)	
Unmarried	11 (0.97%)	0 (0.00%)	11 (1.15%)	
Widowed	160 (14.06%)	21 (11.60%)	139 (14.52%)	
Hukou, n (%)				0.709
Town	314 (27.59%)	52 (28.73%)	262 (27.38%)	
Village	824 (72.41%)	129 (71.27%)	695 (72.62%)	
Smoking, n (%)				0.781
No	852 (74.87%)	137 (75.69%)	715 (74.71%)	
Yes	286 (25.13%)	44 (24.31%)	242 (25.29%)	
Drinking, n (%)				0.418
No	866 (76.10%)	142 (78.45%)	724 (75.65%)	
Yes	272 (23.90%)	39 (21.55%)	233 (24.35%)	
Hypertension, n (%)				0.003
No	454 (39.89%)	54 (29.83%)	400 (41.80%)	
Yes	684 (60.11%)	127 (70.17%)	557 (58.20%)	
Malignant tumor, n (%)				0.015
No	1,124 (98.77%)	175 (96.69%)	949 (99.16%)	
Yes	14 (1.23%)	6 (3.31%)	8 (0.84%)	
Liver disease, n (%)				0.971
No	1,057 (92.88%)	168 (92.82%)	889 (92.89%)	
Yes	81 (7.12%)	13 (7.18%)	68 (7.11%)	
Lung diseases, n (%)				0.415
No	905 (79.53%)	148 (81.77%)	757 (79.10%)	
Yes	233 (20.47%)	33 (18.23%)	200 (20.90%)	
Kidney diease, n (%)				0.809
No	981 (86.20%)	155 (85.64%)	826 (86.31%)	
Yes	157 (13.80%)	26 (14.36%)	131 (13.69%)	
Arthritis or Rheumatism, n (%)				0.672
No	576 (50.62%)	89 (49.17%)	487 (50.89%)	
Yes	562 (49.38%)	92 (50.83%)	470 (49.11%)	
BMI, Median (Q1, Q3)	23.88 (21.41, 26.62)	25.53 (23.35, 28.04)	23.53 (21.20, 26.21)	<0.001
TC, Median (Q1, Q3)	191.17 (169.33, 215.72)	196.78 (172.04, 217.66)	189.82 (169.33, 215.34)	0.276
TG, Median (Q1, Q3)	113.28 (80.54, 166.38)	131.87 (94.70, 206.21)	108.86 (79.65, 161.96)	<0.001
LDL, Median (Q1, Q3)	116.37 (94.72, 139.18)	115.59 (90.85, 139.95)	116.37 (95.88, 138.79)	0.636
HDL, Median (Q1, Q3)	47.94 (39.43, 58.76)	43.69 (35.95, 52.19)	49.10 (39.82, 59.54)	<0.001
UA, Median (Q1, Q3)	4.30 (3.55, 5.15)	4.62 (3.82, 5.21)	4.23 (3.53, 5.11)	0.004
FPG, Median (Q1, Q3)	102.06 (94.68, 111.96)	109.26 (100.08, 119.88)	101.34 (93.78, 110.34)	<0.001
TyG, Median (Q1, Q3)	8.66 (8.28, 9.11)	8.89 (8.50, 9.35)	8.61 (8.24, 9.09)	<0.001
CHG, Median (Q1, Q3)	5.99 (5.74, 6.28)	6.19 (5.85, 6.48)	5.96 (5.72, 6.23)	<0.001

### 3.2 Association of TyG and CHG with incident diabetes in patients with major adverse cardiovascular events

The results of Cox proportional hazards regression analyses are presented in **Table 2**. In the CHARLS dataset, TyG was significantly and positively associated with the risk of incident diabetes. For each 1-standard deviation (SD) increase in TyG, the diabetes risk increased by 46% in the unadjusted model (HR = 1.46, 95% CI: 1.28–1.68), by 32% in the partially adjusted model (HR = 1.32, 95% CI: 1.14–1.52), and by 30% in the fully adjusted model (HR = 1.30, 95% CI: 1.12–1.50; all p<0.001). Analysis by quartiles showed an increasing trend in diabetes risk with higher TyG levels. CHG was also significantly associated with the risk of incident diabetes. For each 1-SD increase, the diabetes risk increased by 1.32-fold in the fully adjusted model (HR = 1.32, 95% CI: 1.14–1.5; p<0.001). Quartile analysis revealed a clear dose-response relationship (P for trend<0.001).

**Table 2:** Association between TyG, CHG, and the risk of developing diabetes in patients with major adverse cardiovascular events.

Variables	Model 1		Model 2		Model 3	
	HR(95%CI)	*p*	HR(95%CI)	*p*	HR(95%CI)	*p*
CHARLS dataset						
TyG (standardized)	1.46 (1.28–1.68)	<0.001	1.32 (1.14–1.52)	<0.001	1.30 (1.12–1.50)	<0.001
TyG						
Q1						
Q2	1.59 (0.96–2.63)	0.069	1.54 (0.93–2.54)	0.094	1.53 (0.92–2.53)	0.100
Q3	2.18 (1.36–3.52)	0.001	1.91 (1.18–3.11)	0.008	1.83 (1.13–2.97)	0.015
Q4	2.71 (1.71–4.30)	<0.001	2.09 (1.31–3.36)	0.002	2.01 (1.25–3.23)	0.004
P for trend		<0.001		0.002		0.003
CHG (standardized)	1.49 (1.30–1.71)	<0.001	1.33 (1.15–1.54)	<0.001	1.32 (1.14–1.53)	<0.001
CHG						
Q1						
Q2	1.15 (0.70–1.88)	0.588	1.02 (0.62–1.67)	0.953	0.99 (0.60–1.63)	0.959
Q3	1.41 (0.88–2.26)	0.154	1.15 (0.71–1.87)	0.557	1.15 (0.71–1.86)	0.572
Q4	2.87 (1.87–4.40)	<0.001	2.08 (1.33–3.26)	0.001	2.06 (1.31–3.22)	0.002
P for trend		<0.001		<0.001		<0.001
CHSY dataset						
TyG (standardized)	1.49 (1.22–1.83)	<0.001	1.47 (1.20–1.80)	<0.001	1.50 (1.22–1.85)	<0.001
TyG						
Q1						
Q2	1.69 (0.74–3.86)	0.214	1.66 (0.72–3.82)	0.231	1.54 (0.67–3.55)	0.315
Q3	1.58 (0.69–3.62)	0.279	1.68 (0.73–3.86)	0.224	1.70 (0.73–3.95)	0.215
Q4	3.82 (1.82–8.00)	<0.001	3.86 (1.82–8.15)	<0.001	4.09 (1.92–8.73)	<0.001
P for trend		<0.001		<0.001		<0.001
CHG (standardized)	1.59 (1.29–1.94)	<0.001	1.62 (1.32–2.00)	<0.001	1.63 (1.33–2.02)	<0.001
CHG						
Q1						
Q2	0.63 (0.26–1.51)	0.300	0.62 (0.25–1.49)	0.283	0.62 (0.25–1.52)	0.296
Q3	1.33 (0.65–2.74)	0.440	1.28 (0.62–2.65)	0.506	1.34 (0.64–2.81)	0.435
Q4	2.69 (1.41–5.11)	0.003	2.88 (1.50–5.53)	0.001	3.09 (1.58–6.05)	<0.001
P for trend		<0.001		<0.001		<0.001

CI, Confidence Interval; HR, Hazard Ratio; Model 1: no covariates were adjusted; Model 2: adjusted for Age, Gender, Marital, Hukou, Smoking, Drinking, and BMI; Model 3: adjusted for Age, Gender, Marital, Hukou, Smoking, Drinking, BMI, Hypertension, Malignant tumor, Lung diseases, Liver diseases, Kidney diseases, and Arthritis or Rheumatism.

In the CHSY dataset, consistent results were observed for both TyG and CHG. For each 1-SD increase in TyG, the diabetes risk increased by 1.50-fold in the fully adjusted model (HR = 1.50, 95% CI: 1.22-1.85). The fully adjusted HR for each 1-SD increase in CHG was 1.63 (95% CI: 1.33–2.02). Trend tests were statistically significant for both (**Table 2**). All models satisfied the proportional hazards assumption (**Supplementary Figures 4**, **5**).

### 3.3 Kaplan-Meier survival curves

Kaplan-Meier curves demonstrated that higher levels of TyG and CHG were associated with higher cumulative incidence of incident diabetes in both the CHARLS and CHSY datasets (**Supplementary Figure 6**). The high-level groups exhibited a higher risk of events early in the follow-up period, and the differences between groups further widened over time (log-rank p<0.001).

### 3.4 Restricted cubic spline analyses

**Figure 2:**
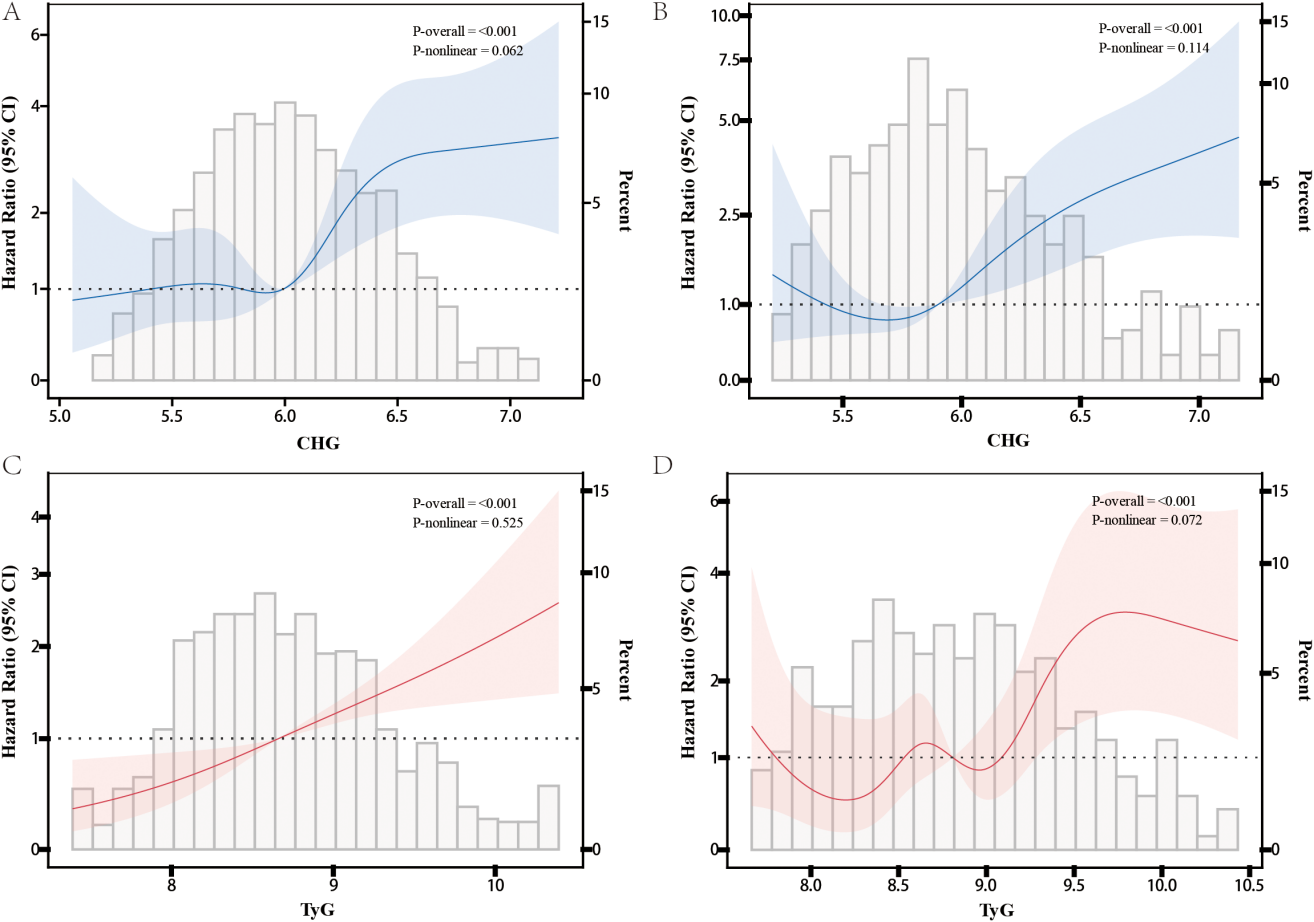
Restricted cubic spline analysis of diabetes risk in patients experiencing major adverse cardiovascular events (MACE) based on TyG and CHG indicators. **(A)** CHG in the CHARLS dataset, **(B)** CHG in the CHSY dataset, **(C)** TyG in the CHARLS dataset, **(D)** TyG in the CHSY dataset.

Restricted cubic spline (RCS) analyses showed that in both datasets, CHG exhibited a stable linear positive association with the risk of incident diabetes, with no significant nonlinear threshold effect observed (**Figures 2A, B**). TyG also demonstrated an overall linear increasing relationship, with only marginal nonlinear trends observed in some models (**Figures 2C, D**). The number of knots for each RCS model was selected based on the lowest Akaike information criterion (AIC), as detailed in **Supplementary Table 4**.

### 3.5 Robustness analyses

Stratified analyses revealed that the positive associations between TyG, CHG and incident diabetes risk were consistent in direction across various subgroups defined by sex, age, and hypertension status (**Supplementary Figures 7**-**9**; **Supplementary Tables 5**-**7**). To evaluate the robustness of the study findings, four sensitivity analyses were conducted within the CHARLS dataset. First, To assess whether class imbalance affects the discriminative ability of the indicators, a balanced analysis was conducted within a classification framework using the SMOTE. ROC curves were generated, and AUC values were compared. Second, a complete-case analysis was employed to verify the results without imputation. Third, multivariable logistic regression was used as a suitable alternative to the Cox model. The fourth key analysis was designed to directly address the issue of mathematical coupling (over-adjustment) by constructing an adjusted model (Model 4) that included all metabolic indicators (BMI, TC, TG, LDL-C, HDL-C, UA) in addition to all covariates from Model 3. The results of all four sensitivity analyses were highly consistent with the primary analysis, further validating the robustness of TyG and CHG as independent predictors of diabetes (see **Supplementary Tables 8**-**11**).

### 3.6 Predictive performance and interpretability

**Figure 3:**
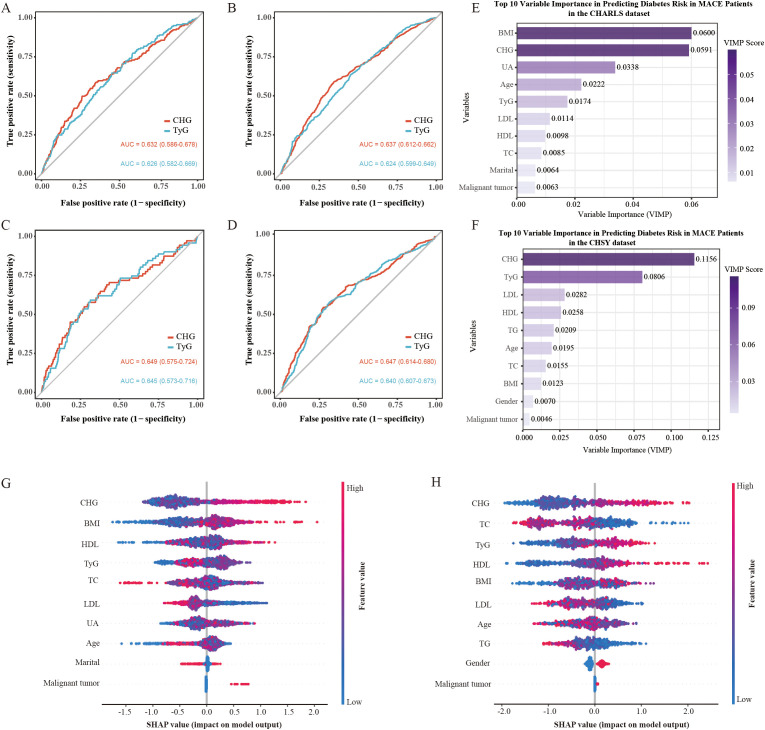
ROC curves, variable importance ranking, and SHAP analysis for the risk of diabetes mellitus development based on CHG, TyG, and major adverse cardiovascular events (MACE). (A–D) display the ROC curves for **(A)** the unbalanced CHARLS dataset, **(B)** the balanced CHARLS dataset, **(C)** the unbalanced CHSY dataset, and **(D)** the balanced CHSY dataset, respectively. (E, F) illustrate the top ten variables in importance for predicting diabetes risk in MACE patients: **(E)** the CHARLS dataset and **(F)** the CHSY dataset. (G, H) present the SHAP summary plots ranking feature importance: **(G)** the CHARLS dataset and **(H)** the CHSY dataset.

ROC curve analysis showed that in both CHARLS and CHSY, the AUC for CHG in predicting incident diabetes was slightly higher than that for TyG (**Figures 3A–D**). To further evaluate the practical value of the models, their overall accuracy and calibration were assessed in CHARLS database. The model based on the CHG index exhibited a slightly lower Brier score (0.1297) compared to the model based on TyG (0.1301). Furthermore, goodness-of-fit tests suggested that both indices possessed acceptable calibration, with the CHG model demonstrating a more balanced fit (Chi-square P = 0.343) than the TyG model (P = 0.890) (**Supplementary Table 12**). Analyses using Random Forest and eXtreme Gradient Boosting (XGBoost) models indicated that the feature importance and SHapley Additive exPlanations (SHAP) contribution of CHG were higher than those of TyG and superior to several traditional metabolic indicators (**Figures 3E–H**).

## 4 Discussion

This study systematically evaluated the value of the CHG and TyG indices in predicting the risk of new-onset diabetes in a MACE population, utilizing two independent prospective cohorts with complementary follow-up methods from different sources. Our results demonstrated that both indices showed a stable, linear, positive correlation with diabetes incidence, with good consistency across various statistical models and machine learning frameworks. More notably, CHG outperformed TyG across risk discrimination, model performance, and feature contribution. This finding suggests that CHG may capture metabolic information beyond the scope of traditional insulin resistance indicators, thereby exhibiting higher sensitivity and specificity in predicting metabolic progression following a cardiovascular event.

TyG has long been regarded as a convenient surrogate marker of insulin resistance, and its predictive value for metabolic diseases has been validated across diverse populations (33, 34). This study also reaffirmed its robust performance in the MACE population. However, within the specific metabolic milieu following a cardiovascular event, an interaction based solely on glucose and triglycerides might not entirely reflect the multidimensional panorama of metabolic remodeling (35, 36). By incorporating TC and HDL-C into its calculation, the biological connotation of CHG more closely aligns with the integrated pathological process of “cholesterol–lipoprotein function–inflammation–insulin resistance,” which is likely the core reason for its superiority over TyG (37, 38).

From a mechanistic perspective, the post-cardiovascular event metabolic environment is highly complex, characterized by persistent inflammatory activation, impaired lipoprotein function, increased hepatic metabolic stress, and progressive dysregulation of insulin signaling pathways (39–41). Elevated TC and reduced HDL-C not only reflect the atherosclerotic burden but are also associated with oxidative stress, immune responses, and impaired hepatic lipid export, all of which can promote insulin resistance through multiple pathways (42, 43). It is particularly noteworthy that, in patients with cardiovascular events, the anti-inflammatory and antioxidant functions of HDL are often compromised, manifesting as alterations in particle size, proteome remodeling, and decreased reverse cholesterol transport efficiency (44). These functional changes not only weaken cardiovascular protection but also promote dysregulation of glucose metabolism by affecting insulin receptor signaling, β-cell stress response, and hepatic fat accumulation, among other pathways (45). Therefore, we hypothesize that the inclusion of the HDL component in the CHG index may link it to “functional HDL abnormalities,” which some studies suggest may better reflect metabolic risk than HDL levels alone.

Furthermore, a tight bidirectional association exists between cholesterol metabolism and hepatic insulin resistance (46). Increased hepatic lipotoxicity, intracellular cholesterol accumulation in hepatocytes, and impaired lipoprotein processing can all impair the IRS-1/PI3K/Akt pathway, thereby accelerating the progression of glucose metabolism disorders (47, 48). Based on this, we postulate that the CHG index may indirectly reflect hepatic metabolic stress related to cholesterol handling and lipoprotein secretion, a dimension not directly captured by the TyG index. Given that the liver is considered a key target organ for metabolic remodeling after a cardiovascular event, the superior performance of the CHG index in this context might be partially explained by this mechanism.

Stratified analyses in this study further revealed heterogeneity in metabolic risk across different subpopulations. The association between CHG and diabetes risk was observed to be more pronounced in females, younger individuals, and those with hypertension. Females exhibit a greater decline in lipoprotein function after cardiovascular events, and hormonal changes can affect HDL’s anti-inflammatory capacity, potentially making CHG more sensitive to metabolic impairment (49, 50). In younger populations, despite greater metabolic adaptive capacity, metabolic fluctuations are also larger, making the linear relationship between CHG and risk more readily detectable. In hypertensive patients, mechanisms such as sympathetic activation, RAAS hyperactivity, and endothelial dysfunction contribute to a tighter “inflammation–metabolism–vasculature” linkage, which may amplify the association between CHG and diabetes risk (51). These findings suggest that the utility of the CHG index as a metabolic risk marker is not limited to specific patient subsets and support its general applicability in the post-MACE population.

From a clinical standpoint, the CHG index, a derived parameter based on routine laboratory measures, offers advantages such as ease of accessibility, low cost, and strong reproducibility. In the MACE population, traditional metabolic risk assessments often fail to fully capture the complexity of metabolic remodeling following a cardiovascular event. As a composite index, CHG holds promise as a practical tool for follow-up management to identify patients on a trajectory of metabolic deterioration, thereby providing a basis for earlier interventions, including lifestyle intensification, medication optimization, and metabolic-cardiovascular management strategies. More importantly, CHG’s performance suggests that future metabolic risk assessment methods may need to extend beyond traditional single metabolic dimensions and incorporate information on lipoprotein functional status, inflammatory responses, hepatic lipid handling, and other aspects. Its potential clinical utility lies in serving as a composite risk-stratification indicator during follow-up. For instance, an elevated CHG level may indicate a pattern of metabolic disorder—even when individual lipid or glucose parameters are controlled—suggesting heightened insulin resistance and residual inflammatory lipid risk. Such identification could prompt clinicians to intensify lifestyle counseling, initiate earlier and more frequent glucose monitoring, or consider the use of cardiometabolic medications proven to offer dual benefits in secondary prevention and diabetes risk reduction. Compared with the well-established TyG index, the CHG index is not intended to replace it but rather to provide supplementary information. Integrating the CHG index into electronic health records for automated calculation could facilitate population-level risk screening. However, it must be emphasized that the precise clinical utility of the CHG index in guiding specific interventions, as well as its optimal cutoff value, requires validation through prospective interventional studies. Future research should also explore the dynamic response of the CHG index to treatment and its association with cardiovascular outcomes.

Although this study provides multiple lines of evidence supporting the predictive value of CHG, certain limitations must be acknowledged. First, despite using two distinct data sources, the potential influence of population differences and residual confounding cannot be entirely excluded. Second, the lack of gold-standard insulin sensitivity measurements and indicators of lipoprotein function precludes direct validation of the biological mechanisms underlying CHG. Third, in our primary analysis of the population following adverse cardiovascular events, a competing risk model that explicitly accounted for death was not incorporated. Although this model is more relevant for estimating absolute cumulative occurrence rates than for the comparative analyses of biomarker relative strength and predictive value conducted in this study, it remains a profound methodological consideration. Future research could employ competing risk models to quantify the net risk of diabetes in this context. Furthermore, this study was primarily based on a Chinese population, and its applicability to other ethnicities, dietary patterns, and cardiovascular risk backgrounds requires further investigation.

## 5 Conclusion

This study systematically evaluated the predictive value of the CHG and TyG indices for new-onset diabetes among patients with MACE, using two independent population datasets: CHARLS and CHSY. The results demonstrated a robust and consistent association between the CHG index and the risk of new-onset diabetes. Its discriminatory ability was comparable to that of the well-established TyG index, while it showed a slight advantage in overall predictive accuracy and model calibration. These findings suggest that the CHG index possesses strong clinical utility for identifying diabetes risk in the post-cardiovascular event population.

## Data Availability

The raw data supporting the conclusions of this article will be made available by the authors, without undue reservation. Requests to access these datasets should be directed to JUN Liu, junliu1314520@163.com.

## Supplementary material

Supplementary Figure 1 Lollipop chart of missing rates for study variables. **(A)** Missing rates of variables in the CHARLS dataset, **(B)** Missing rates of variables in the CHSY dataset, **(C)** Missing Variable Imputation Density Map in CHARLS Database and **(D)** Missing Variable Imputation Density Map in CHSY dataset.

Supplementary Figure 2 Variable correlation analysis plots. **(A)** Correlation Analysis of Variables in the CHARLS dataset and **(B)** Correlation Analysis of Variables in the CHSY dataset.

Supplementary Figure 3 Collinearity analysis plots. **(A)** Collinearity analysis of variables in the CHARLS dataset, **(B)** Collinearity analysis of variables in the CHSY dataset.

Supplementary Figure 4 CHG residual test plot. **(A)** Residual plots of CHG and other covariates in the CHARLS database and **(B)** Residual plots of CHG and other covariates in the CHSY database.

Supplementary Figure 5 TyG residual test plot. **(A)** Residual plots of TyG and other covariates in the CHARLS database and **(B)** Residual plots of TyG and other covariates in the CHSY database.

Supplementary Figure 6 Cumulative incidence curves for diabetes based on the TyG and CHG indices in patients with major adverse cardiovascular events (MACE). Panels **(A–D)** present the Kaplan-Meier curves for the cumulative incidence of diabetes, stratified as follows: **(A)** Kaplan-Meier curves for diabetes risk in patients with major adverse cardiovascular events, stratified by CHG index categories in the CHARLS dataset, **(B)** Kaplan-Meier curves for diabetes risk in patients with major adverse cardiovascular events, stratified by CHG index categories in the CHSY dataset, **(C)** Kaplan-Meier curves for diabetes risk in patients with major adverse cardiovascular events, stratified by TyG index categories in the CHARLS dataset, **(D)** Kaplan-Meier curves for diabetes risk in patients with major adverse cardiovascular events, stratified by TyG index categories in the CHSY dataset.

Supplementary Figure 7 Analysis of the association between CHG, TyG, and diabetes risk in patients with major adverse cardiovascular events (MACE), stratified by sex. Panels A -D present the sex-stratified cumulative incidence Kaplan-Meier curves from the CHARLS dataset: **(A)** Kaplan-Meier survival curve for CHG in the male population, **(B)** Kaplan-Meier survival curve for TyG in the male population, **(C)** Kaplan-Meier survival curve for CHG in the female population, and **(D)** Kaplan-Meier survival curve for TyG in the female population. Panels E -H present the sex-stratified restricted cubic spline (RCS) curves from the CHARLS dataset: **(E)** RCS analysis depicting the association between CHG and the risk of the outcome event in the male population, **(F)** RCS analysis depicting the association between TyG and the risk of the outcome event in the male population, **(G)** RCS analysis depicting the association between CHG and the risk of the outcome event in the female population, and **(H)** RCS analysis depicting the association between TyG and the risk of the outcome event in the female population.

Supplementary Figure 8 Analysis of the association between CHG, TyG, and diabetes risk in patients with major adverse cardiovascular events (MACE), stratified by age. Panels A–D present the age-stratified cumulative incidence Kaplan-Meier curves from the CHARLS dataset: **(A)** Kaplan-Meier survival curve for CHG in younger age group, **(B)** Kaplan-Meier survival curve for TyG in the younger age group, **(C)** Kaplan-Meier survival curve for CHG in the older age group, and **(D)** Kaplan-Meier survival curve for TyG in the older age group. Panels E–H present the age-stratified restricted cubic spline (RCS) curves from the CHARLS dataset: **(E)** RCS analysis depicting the association between CHG and the risk of the outcome event in the younger age group, **(F)** RCS analysis depicting the association between TyG and the risk of the outcome event in the younger age group, **(G)** RCS analysis depicting the association between CHG and the risk of the outcome event in the older age group, and **(H)** RCS analysis depicting the association between TyG and the risk of the outcome event in the older age group.

Supplementary Figure 9 Analysis of the association between CHG, TyG, and diabetes risk in patients with major adverse cardiovascular events (MACE), stratified by hypertension status. Panels A–D present the hypertension status-stratified cumulative incidence Kaplan-Meier curves from the CHARLS dataset: **(A)** Kaplan-Meier survival curve for CHG in the hypertensive population, **(B)** Kaplan-Meier survival curve for TyG in the hypertensive population, **(C)** Kaplan-Meier survival curve for CHG in the non-hypertensive population, and **(D)** Kaplan-Meier survival curve for TyG in the non-hypertensive population. Panels E -H present the hypertension status-stratified restricted cubic spline (RCS) curves from the CHARLS dataset: **(E)** RCS analysis depicting the association between CHG and the risk of the outcome event in the hypertensive population, **(F)** RCS analysis depicting the association between TyG and the risk of the outcome event in the hypertensive population, **(G)** RCS analysis depicting the association between CHG and the risk of the outcome event in the non-hypertensive population, and **(H)** RCS analysis depicting the association between TyG and the risk of the outcome event in the non-hypertensive population.

Supplementary Table 1 Description of the study variables.

Supplementary Table 2 Results of normality tests.

Supplementary Table 3 Patient demographics and baseline characteristics in the CHSY dataset.

Supplementary Table 4 AIC value of different knots in RCS analysis.

Supplementary Table 5 Association between TyG, CHG, and the risk of developing diabetes in patients with Major Adverse Cardiovascular Events by gender.

Supplementary Table 6 Association between TyG, CHG, and the risk of developing diabetes in patients with Major Adverse Cardiovascular Events by age.

Supplementary Table 7 Association between TyG, CHG, and the risk of developing diabetes in patients with Major Adverse Cardiovascular Events by Hypertension.

Supplementary Table 8 Sensitivity Analysis of the Association Between TyG, CHG, and Diabetes Risk in Patients with Major Adverse Cardiovascular Events in the SMOTE dataset.

Supplementary Table 9 Sensitivity analysis following direct imputation of missing data in the CHARLS dataset.

Supplementary Table 10 Logistic Regression Analysis in the CHARLS dataset.

Supplementary Table 11 Association of the TyG and CHG indices with incident diabetes risk in MACE patients: Main analysis and sensitivity analysis with adjustment for constituent metabolic components.

Supplementary Table 12 Discrimination and Calibration Performance of the TyG and CHG Models in Predicting Diabetes Risk among MACE Patients.
