# Supplementary material for: Beyond the triglyceride-glucose index, the cholesterol- high-density lipoprotein -glucose index as a superior predictor for diabetes risk in patients with major adverse cardiovascular events: dual evidence from the CHARLS database and real-world data
Source: Front Endocrinol (Lausanne). 2026 Mar 12;17:1797342. doi: 10.3389/fendo.2026.1797342 (PMC13019095; doi:10.3389/fendo.2026.1797342)
Supplement: Supplementary Figure 1 — Lollipop chart of missing rates for study variables. (A) Missing rates of variables in the CHARLS dataset, (B) Missing rates of variables in the CHSY dataset, (C) Missing Variable Imputation Density Map in CHARLS Database and (D) Missing Variable Imputation Density Map in CHSY dataset. [file DataSheet1.zip › Supplementary Table 1.docx]

Supplementary Table 1. Description of the study variables.

| **SN** | **Predictors** | **Discription** | **Types** | **Values** |
| --- | --- | --- | --- | --- |
| 1 | Age | CHARLS dataset: The age of participants in the CHARLS dataset was determined by calculating the difference between 2011 and the respondent's year of birth.  CHSY dataset: The age of participants in the CHSY dataset was recorded in the HIS database of the Central Hospital of Shaoyang. | Continuous | CHARLS dataset: 38-90  CHSY dataset: 33-94 |
| 2 | Gender | Sex of the patient | Categorical | 1: Male  2: Female |
| 3 | Drinking | CHARLS dataset: If the respondent answers that they do not drink anything (including beer, wine, or liquor), they are defined as "non-drinking"; if they answer that they drink more than once a month or less than once a month, they are defined as "drinking."  CHSY dataset: History of alcohol consumption derived from medical records in the HIS system of the Central Hospital of Shaoyang. | Categorical | 0: No  1: Yes |
| 4 | Smoking | CHARLS Dataset: Smoking status was categorized into two groups based on respondents' answers to the questionnaire. Participants who reported never having smoked (including cigarettes, pipes, or chewing tobacco) and those who reported having smoked in the past but had since quit were classified as "non-smokers." Individuals who confirmed current smoking habits were classified as "smokers."  CHSY Dataset: Smoking status was obtained from the medical records in the Hospital Information System (HIS) database of the Central Hospital of Shaoyang. | Categorical | 0: No  1: Yes |
| 5 | Hypertension  (HTN) | CHARLS Dataset: Individuals were classified as "non-hypertensive" if their systolic blood pressure was below 140 mmHg and diastolic blood pressure below 90 mmHg, they had not been diagnosed with hypertension, and had not been subjected to any antihypertensive treatment. Otherwise, they were categorized into the "hypertensive" group.  CHSY Dataset: History of chronic diseases was obtained from the medical records within the Hospital Information System (HIS) database of the Central Hospital of Shaoyang. | Categorical | 0: No  1: Yes |
| 6 | Malignant tumor | CHARLS Dataset: Data were derived from questionnaire item DA007(4): "Has a doctor ever told you that you have a malignant tumor such as cancer (excluding mild skin cancer)?" Affirmative responses (coded as 1) were classified as having a history of malignant tumors, while negative responses (coded as 2) were classified as having no such history.  CHSY Dataset: History of chronic diseases was obtained from the medical records within the Hospital Information System (HIS) database of the Central Hospital of Shaoyang. | Categorical | 0: No  1: Yes |
| 7 | Lung diseases | CHARLS Dataset: Data were derived from questionnaire item DA007(5): "Has a doctor ever told you that you have chronic lung diseases, such as chronic bronchitis, emphysema (excluding tumors, or cancer)?" Affirmative responses (coded as 1) were classified as having a history of chronic lung diseases, while negative responses (coded as 2) were classified as having no such history.  CHSY Dataset: History of chronic diseases was obtained from the medical records within the Hospital Information System (HIS) database of the Central Hospital of Shaoyang. | Categorical | 0: No  1: Yes |
| 8 | Liver diseases | CHARLS Dataset: Data were derived from questionnaire item DA007(6): "Has a doctor ever told you that you have liver disease (except fatty liver, tumors, and cancer)?" Affirmative responses (coded as 1) were classified as having a history of liver disease, while negative responses (coded as 2) were classified as having no such history.  CHSY Dataset: History of chronic diseases was obtained from the medical records within the Hospital Information System (HIS) database of the Central Hospital of Shaoyang. | Categorical | 0: No  1: Yes |
| 9 | Kidney diseases | CHARLS Dataset: Data were derived from questionnaire item DA007(9): "Has a doctor ever told you that you have kidney disease (except for tumor or cancer)?" Affirmative responses (coded as 1) were classified as having a history of kidney disease, while negative responses (coded as 2) were classified as having no such history.  CHSY Dataset: History of chronic diseases was obtained from the medical records within the Hospital Information System (HIS) database of the Central Hospital of Shaoyang. | Categorical | 0: No  1: Yes |
| 10 | Arthritis or Rheumatism | CHARLS Dataset: Data were derived from questionnaire item DA007(13): "Has a doctor ever told you that you have arthritis or rheumatism?" Affirmative responses (coded as 1) were classified as having a history of arthritis or rheumatism, while negative responses (coded as 2) were classified as having no such history.  CHSY Dataset: History of chronic diseases was obtained from the medical records within the Hospital Information System (HIS) database of the Central Hospital of Shaoyang. | Categorical | 0: No  1: Yes |
| 11 | Body mass index (BMI) | BMI is a widely utilized metric for assessing the ratio of an individual's weight to height. It is calculated by dividing the individual's weight in kilograms by the square of their height in meters. | Continuous | CHARLS dataset: 16.057-37.099  CHSY dataset: 16.167-39.013 |
| 12 | Marital | Marital status is categorized into four groups: married, divorced, widowed, and unmarried, based on the responses to the question "What is your current marital status?" | Categorical | 1: Married  2: Divorced  3: Widowed  4: Unmarried |
| 13 | Hukou | Hukou is categorized into two levels on the basis of the response to "What is the current hukou status": village and town. | Categorical | 1: Village  2: Town |
| 14 | TG | Triglycerides (mg/dl) | Continuous | CHARLS dataset: 40.710-497.273  CHSY dataset: 41.973-720.845 |
| 15 | LDL | Low density lipoprotein cholesterol (mg/dl) | Continuous | CHARLS dataset: 42.955-216.210  CHSY dataset: 31.129-174.712 |
| 16 | HDL | High density lipoprotein cholesterol (mg/dl) | Continuous | CHARLS dataset: 22.179-94.102  CHSY dataset: 24.019-83.069 |
| 17 | UA | Uric Acid (mg/dl) | Continuous | CHARLS dataset: 2.033-8.197  CHSY dataset: 4.025-6.535 |
| 18 | FPG | Fasting blood glucose (mg/dl) | Continuous | CHARLS dataset: 56.493-180.794  CHSY dataset: 70.301-315.424 |
| 19 | TyG | Triglyceride-glucose Index | Continuous | CHARLS dataset: 7.389-10.392  CHSY dataset: 7.594-11.072 |
| 20 | CHG | Cholesterol-HDL-glucose index | Continuous | CHARLS dataset: 5.057-7.215  CHSY dataset: 5.072-7.224 |
