# Supplementary material for: Beyond the triglyceride-glucose index, the cholesterol- high-density lipoprotein -glucose index as a superior predictor for diabetes risk in patients with major adverse cardiovascular events: dual evidence from the CHARLS database and real-world data
Source: Front Endocrinol (Lausanne). 2026 Mar 12;17:1797342. doi: 10.3389/fendo.2026.1797342 (PMC13019095; doi:10.3389/fendo.2026.1797342)
Supplement: Supplementary Figure 1 — Lollipop chart of missing rates for study variables. (A) Missing rates of variables in the CHARLS dataset, (B) Missing rates of variables in the CHSY dataset, (C) Missing Variable Imputation Density Map in CHARLS Database and (D) Missing Variable Imputation Density Map in CHSY dataset. [file DataSheet1.zip › Supplementary Table 11.docx]

Supplementary Table 11 Association of the TyG and CHG indices with incident diabetes risk in MACE patients: Main analysis and sensitivity analysis with adjustment for constituent metabolic components.

| **Variables** | **Model 1** | | **Model 2** | | **Model 3** | | **Model 4** | |
| --- | --- | --- | --- | --- | --- | --- | --- | --- |
|  | **HR(95%CI)** | ***p*** | **HR(95%CI)** | ***p*** | **HR(95%CI)** | ***p*** | **HR(95%CI)** | ***p*** |
| **CHARLS dataset** |  |  |  |  |  |  |  |  |
| **TyG (standardized)** | 1.46 (1.28–1.68) | <0.001 | 1.32 (1.14–1.52) | <0.001 | 1.30 (1.12–1.50) | <0.001 | 2.41 (1.65–3.53) | <0.001 |
| **TyG** |  |  |  |  |  |  |  |  |
| Q1 |  |  |  |  |  |  |  |  |
| Q2 | 1.59 (0.96–2.63) | 0.069 | 1.54 (0.93–2.54) | 0.094 | 1.53 (0.92–2.53) | 0.100 | 1.55 (0.92–2.61) | 0.098 |
| Q3 | 2.18 (1.36–3.52) | 0.001 | 1.91 (1.18–3.11) | 0.008 | 1.83 (1.13–2.97) | 0.015 | 1.96 (1.14–3.38) | 0.016 |
| Q4 | 2.71 (1.71–4.30) | <0.001 | 2.09 (1.31–3.36) | 0.002 | 2.01 (1.25–3.23) | 0.004 | 2.25 (1.12–4.51) | 0.022 |
| P for trend | 1.46 (1.28–1.68) | <0.001 | 1.32 (1.14–1.52) | <0.001 | 1.30 (1.12–1.50) | <0.001 |  | 0.015 |
| **CHG (standardized)** | 1.49 (1.30–1.71) | <0.001 | 1.33 (1.15–1.54) | <0.001 | 1.32 (1.14–1.53) | <0.001 | 2.17 (1.56–3.01) | <0.001 |
| **CHG** |  |  |  |  |  |  |  |  |
| Q1 |  |  |  |  |  |  |  |  |
| Q2 | 1.15 (0.70–1.88) | 0.588 | 1.02 (0.62–1.67) | 0.953 | 0.99 (0.60–1.63) | 0.959 | 1.26 (0.73–2.17) | 0.414 |
| Q3 | 1.41 (0.88–2.26) | 0.154 | 1.15 (0.71–1.87) | 0.557 | 1.15 (0.71–1.86) | 0.572 | 1.84 (0.98–3.44) | 0.056 |
| Q4 | 2.87 (1.87–4.40) | <0.001 | 2.08 (1.33–3.26) | 0.001 | 2.06 (1.31–3.22) | 0.002 | 4.20 (1.99–8.85) | <0.001 |
| P for trend |  | <0.001 |  | <0.001 |  | <0.001 |  | <0.001 |
| **CHSY dataset** |  |  |  |  |  |  |  |  |
| **TyG (standardized)** | 1.49 (1.22–1.83) | <0.001 | 1.47 (1.20–1.80) | <0.001 | 1.50 (1.22–1.85) | <0.001 | 3.25 (2.19–4.82) | <0.001 |
| **TyG** |  |  |  |  |  |  |  |  |
| Q1 |  |  |  |  |  |  |  |  |
| Q2 | 1.69 (0.74–3.86) | 0.214 | 1.66 (0.72–3.82) | 0.231 | 1.54 (0.67–3.55) | 0.315 | 1.81 (0.78–4.23) | 0.169 |
| Q3 | 1.58 (0.69–3.62) | 0.279 | 1.68 (0.73–3.86) | 0.224 | 1.70 (0.73–3.95) | 0.215 | 2.37 (0.98–5.73) | 0.055 |
| Q4 | 3.82 (1.82–8.00) | <0.001 | 3.86 (1.82–8.15) | <0.001 | 4.09 (1.92–8.73) | <0.001 | 9.07 (3.62–22.78) | <0.001 |
| P for trend |  | <0.001 |  | <0.001 |  | <0.001 |  | <0.001 |
| **CHG (standardized)** | 1.59 (1.29–1.94) | <0.001 | 1.62 (1.32–2.00) | <0.001 | 1.63 (1.33–2.02) | <0.001 | 2.58 (1.94–3.42) | <0.001 |
| **CHG** |  |  |  |  |  |  |  |  |
| Q1 |  |  |  |  |  |  |  |  |
| Q2 | 0.63 (0.26–1.51) | 0.300 | 0.62 (0.25–1.49) | 0.283 | 0.62 (0.25–1.52) | 0.296 | 1.02 (0.40–2.59) | 0.975 |
| Q3 | 1.33 (0.65–2.74) | 0.440 | 1.28 (0.62–2.65) | 0.506 | 1.34 (0.64–2.81) | 0.435 | 2.54 (1.12–5.76) | 0.026 |
| Q4 | 2.69 (1.41–5.11) | 0.003 | 2.88 (1.50–5.53) | 0.001 | 3.09 (1.58–6.05) | <0.001 | 9.01 (3.74–21.72) | <0.001 |
| P for trend |  | <0.001 |  | <0.001 |  | <0.001 |  | <0.001 |

Abbreviations: CI = Confidence Interval, HR = Hazard Ratio; Model 1: no covariates were adjusted; Model 2: adjusted for Age, Gender, Marital, Hukou, Smoking, Drinking, and BMI; Model 3: adjusted for Age, Gender, Marital, Hukou, Smoking, Drinking, BMI, Hypertension, Malignant tumor, Lung diseases, Liver diseases, Kidney diseases, and Arthritis or Rheumatism; Model 4: adjusted for Age, Gender, Marital, Hukou, Smoking, Drinking, Hypertension, Malignant tumor, Lung diseases, Liver diseases, Kidney diseases, Arthritis or Rheumatism, BMI, TC, TG, LDL, HDL, and UA.
