# Supplementary material for: Beyond the triglyceride-glucose index, the cholesterol- high-density lipoprotein -glucose index as a superior predictor for diabetes risk in patients with major adverse cardiovascular events: dual evidence from the CHARLS database and real-world data
Source: Front Endocrinol (Lausanne). 2026 Mar 12;17:1797342. doi: 10.3389/fendo.2026.1797342 (PMC13019095; doi:10.3389/fendo.2026.1797342)
Supplement: Supplementary Figure 1 — Lollipop chart of missing rates for study variables. (A) Missing rates of variables in the CHARLS dataset, (B) Missing rates of variables in the CHSY dataset, (C) Missing Variable Imputation Density Map in CHARLS Database and (D) Missing Variable Imputation Density Map in CHSY dataset. [file DataSheet1.zip › Supplementary Table 3.docx]

Supplementary Table 3. Patient demographics and baseline characteristics in the CHSY dataset.

| **Characteristic** | **Event** | | | **p-value** |
| --- | --- | --- | --- | --- |
|  | **Overall  N = 614** | **MACE with diabetes  N = 71** | **MACE without diabetes  N = 543** |  |
| **Age, Median (Q1, Q3)** | 66.00 (56.00, 74.00) | 66.00 (57.00, 74.00) | 66.00 (56.00, 74.00) | 0.927 |
| **Gender, n (%)** |  |  |  | 0.234 |
| Female | 237 (38.60%) | 32 (45.07%) | 205 (37.75%) |  |
| Male | 377 (61.40%) | 39 (54.93%) | 338 (62.25%) |  |
| **Marital, n (%)** |  |  |  | 0.887 |
| Divorced | 46 (7.49%) | 4 (5.63%) | 42 (7.73%) |  |
| Married | 490 (79.80%) | 60 (84.51%) | 430 (79.19%) |  |
| Unmarried | 66 (10.75%) | 6 (8.45%) | 60 (11.05%) |  |
| Widowed | 12 (1.95%) | 1 (1.41%) | 11 (2.03%) |  |
| **Hukou, n (%)** |  |  |  | 0.699 |
| Town | 136 (22.15%) | 17 (23.94%) | 119 (21.92%) |  |
| Village | 478 (77.85%) | 54 (76.06%) | 424 (78.08%) |  |
| **Smoking, n (%)** |  |  |  | 0.270 |
| No | 343 (55.86%) | 44 (61.97%) | 299 (55.06%) |  |
| Yes | 271 (44.14%) | 27 (38.03%) | 244 (44.94%) |  |
| **Drinking, n (%)** |  |  |  | 0.058 |
| No | 342 (55.70%) | 47 (66.20%) | 295 (54.33%) |  |
| Yes | 272 (44.30%) | 24 (33.80%) | 248 (45.67%) |  |
| **Hypertension, n (%)** |  |  |  | 0.043 |
| No | 411 (66.94%) | 40 (56.34%) | 371 (68.32%) |  |
| Yes | 203 (33.06%) | 31 (43.66%) | 172 (31.68%) |  |
| **Malignant tumor, n (%)** |  |  |  | 0.376 |
| No | 584 (95.11%) | 66 (92.96%) | 518 (95.40%) |  |
| Yes | 30 (4.89%) | 5 (7.04%) | 25 (4.60%) |  |
| **Liver disease, n (%)** |  |  |  | 0.648 |
| No | 546 (88.93%) | 62 (87.32%) | 484 (89.13%) |  |
| Yes | 68 (11.07%) | 9 (12.68%) | 59 (10.87%) |  |
| **Lung diseases, n (%)** |  |  |  | 0.204 |
| No | 493 (80.29%) | 53 (74.65%) | 440 (81.03%) |  |
| Yes | 121 (19.71%) | 18 (25.35%) | 103 (18.97%) |  |
| **Kidney diease, n (%)** |  |  |  | 0.507 |
| No | 510 (83.06%) | 57 (80.28%) | 453 (83.43%) |  |
| Yes | 104 (16.94%) | 14 (19.72%) | 90 (16.57%) |  |
| **Arthritis or Rheumatism, n (%)** |  |  |  | 0.058 |
| No | 483 (78.66%) | 62 (87.32%) | 421 (77.53%) |  |
| Yes | 131 (21.34%) | 9 (12.68%) | 122 (22.47%) |  |
| **BMI, Median (Q1, Q3)** | 24.53 (20.14, 28.27) | 25.21 (20.05, 28.65) | 24.48 (20.15, 28.17) | 0.467 |
| **TC, Median (Q1, Q3)** | 151.93 (127.19, 184.41) | 143.43 (120.23, 190.98) | 153.09 (127.58, 182.86) | 0.370 |
| **TG, Median (Q1, Q3)** | 116.03 (79.71, 172.71) | 129.31 (82.37, 179.80) | 114.26 (79.71, 171.83) | 0.343 |
| **LDL, Median (Q1, Q3)** | 81.57 (62.63, 104.38) | 75.77 (57.60, 104.38) | 82.35 (63.40, 104.77) | 0.269 |
| **HDL, Median (Q1, Q3)** | 44.46 (37.50, 52.19) | 44.46 (36.73, 52.19) | 44.46 (37.50, 52.58) | 0.838 |
| **UA, Median (Q1, Q3)** | 5.05 (4.61, 5.50) | 5.06 (4.64, 5.53) | 5.05 (4.59, 5.49) | 0.778 |
| **GLU, Median (Q1, Q3)** | 105.15 (92.26, 132.81) | 145.24 (111.90, 179.66) | 103.07 (91.00, 125.60) | <0.001 |
| **TyG, Median (Q1, Q3)** | 8.81 (8.36, 9.28) | 9.22 (8.54, 9.64) | 8.76 (8.33, 9.20) | <0.001 |
| **CHG, Median (Q1, Q3)** | 5.90 (5.66, 6.23) | 6.18 (5.77, 6.54) | 5.88 (5.64, 6.17) | <0.001 |
